# Evaluation of the disease burden of nosocomial infection among inpatients in a cancer hospital based on propensity score matching

**DOI:** 10.3389/fpubh.2025.1572558

**Published:** 2025-07-04

**Authors:** Jiayang Tang, Jiang Yuan, Hui Wang, Qin Huang, Weiwei Yang, Qingqing Tian, Anran Liu, Hailin Zhang, Chunlin Wu

**Affiliations:** ^1^Sichuan Clinical Research Center for Cancer, Sichuan Cancer Hospital and Institute, Sichuan Cancer Center, University of Electronic Science and Technology of China, Chengdu, China; ^2^School of Public Health, Chengdu University of Traditional Chinese Medicine, Chengdu, China

**Keywords:** propensity score matching, nosocomial infection, multidrug-resistant bacterial infections, the disease burden, oncology patients

## Abstract

**Background:**

Oncology patients, as an immunocompromised population, are particularly susceptible to hospital-acquired infections (HAIs). This study aims to establish a scientifically sound model to estimate the disease burden associated with HAIs in oncology patients, providing valuable decision-making support for healthcare systems and public health management.

**Methods:**

Propensity score matching (PSM) was employed. Post-matching permutation tests were applied to compare hospitalization costs and lengths of stay between the HAIs and non-HAI groups, as well as between the multidrug-resistant bacteria (MDRO) infection group and non-MDRO group.

**Results:**

The results indicate that the average hospitalization cost in the HAIs group was 23.7% higher than the non-HAI groups, with a median difference of 12,417.6 CNY. Among various hospitalization expenses, the largest disparity was observed in the cost of Western medications, which had a median difference of 5,453.79 CNY, representing a 54.38% increase. The average length of hospital stay in the HAIs group was 1.33 times that of the control group. For the MDRO infection group, the average hospitalization cost exceeded that of the control group by 56.43%, with an absolute difference of 32,266.62 CNY. Additionally, the average length of hospital stay in the MDRO group was 42.11% longer, extending by 8 days.

**Conclusion:**

Both HAIs and MDRO infections significantly increase hospitalization duration and costs. The resulting disease burden is reflected in the direct escalation of health economic costs and the indirect effects of reduced hospital operational efficiency and heightened strain on the healthcare system. On this basis, we conclude that the funds for the prevention and control of HAIs and MDRO infections should be increased, and the measures for the prevention and control of HAIs should be implemented effectively, so as to reduce the direct and indirect economic burdens caused by HAIs and MDRO infections.

## 1 Introduction

With the advancement of medical science and continuous improvements in diagnostic and therapeutic techniques, the frequency of invasive procedures has increased, making HAIs and MDRO infections a growing focus in ensuring medical safety. Oncology patients, due to their underlying diseases and the immunosuppressive effects of radiotherapy and chemotherapy, often experience weakened or suppressed immune function, rendering them more vulnerable to infections (1–3). Additionally, frequent exposure to invasive medical interventions such as central venous catheterization and urinary catheterization, coupled with repeated hospital admissions for treatment, further predisposes them to HAIs and MDRO infections (4–6).

These infections not only delay disease recovery, prolong hospitalization, and increase medical expenses but also impose significant economic burdens. These burdens manifest not only as direct financial costs to patients and their families (7, 8), but also as reduced bed turnover and diminished hospital efficiency, leading to indirect economic pressures on healthcare systems and society at large (9). Moreover, these challenges exacerbate time and economic costs for both patients and society. Despite these significant impacts, HAIs and MDRO infection cases remain a minority within the overall patient population, and research specifically addressing the disease burden of HAIs and MDRO infections in oncology patients remains limited. To investigate the additional burden posed by these infections in oncology patients, this study employs the PSM method to estimate the increased health economic burden caused by HAIs and MDRO infections.

## 2 Materials and methods

### 2.1 Study sample

This study was conducted at a specialized oncology hospital in western China, used data from discharged patients between May 2023 and May 2024. Patient demographic and clinical information was retrieved from the hospital information system. The inclusion criteria for the study samples are as follows. First of all, the hospitalization period must be between May 2023 and May 2024. Second, identifications must be complete without any missing data, and there should be comprehensive details regarding disease diagnosis and hospitalization costs. For patients with hospital-acquired infections, relevant information must also be available. A total of 558 patients with incomplete information were excluded, resulting in 86,177 study samples who met the inclusion criteria. Ethical approval for the study was obtained from the hospital’s ethics committee. Data on all discharged patients were extracted from the hospital infection management system, including variables such as gender, age, admission and discharge dates, preoperative NLR, surgical status, days of mechanical ventilation, days of central venous catheterization, days of urinary catheterization, total hospitalization costs, and detailed expense categories (e.g., bed fees, nursing fees, medication fees, and diagnostic fees). Additionally, data on HAIs, HAI types, and MDRO infection types were collected.

The criteria for diagnosing HAIs were based on the standards outlined in the Ministry of Health Diagnostic Criteria for Hospital-Acquired Infections (Trial Implementation). The relevant definitions of nosocomial infections in the Diagnostic Criteria for Hospital-Acquired Infections (Trial Implementation) issued by the Ministry of Health. The diagnostic criteria for MDRO infections followed the Technical Guidelines for *the Prevention and Control of Multidrug-Resistant Bacterial Hospital Infections (2011)*, which define MDRO as bacteria resistant to three or more commonly used antimicrobial drug classes that are typically effective against them.

### 2.2 Methods

Statistical processing and analysis were conducted using R version 4.4.1. For quantitative data with a skewed distribution (e.g., hospitalization duration, hospitalization costs), medians were calculated for statistical description. Propensity score matching (PSM) was implemented using the `MatchIt` package, with HAI status as the dependent variable. A nearest-neighbor 1:1 matching was performed for HAI patients and non-HAI patients. Significance was evaluated at *p* < 0.05 and 95% confidence intervals were reported. A cutoff threshold of 0.2 standardized mean differences was applied (10). Post-matching, the ‘Coin’ package in R was used to perform permutation tests to compare groups. Covariates included age, gender, neutrophil-to-lymphocyte ratio (NLR), surgical status, days of mechanical ventilation, days of central venous catheterization, and days of urinary catheterization. The intergroup comparisons were conducted between the HAIs group, the MDRO infection group, and their respective control groups. The outcome variables included total hospitalization costs, hospitalization duration, and categorized costs such as bed fees, nursing fees, and medication costs.

## 3 Results

### 3.1 General and hospital-acquired infection characteristics

The study included 86,177 patients, comprising 40,560 males (47.07%) and 45,617 females (52.93%), with a median age of 56 years [P25: 49, P75: 65, same below]. The length of hospitalization followed a skewed distribution (normality test: *p* < 0.05), with a median of 6 days [4.0, 9.0]. Similarly, hospitalization costs were skewed (normality test: p < 0.05), with a median cost of 8,643.38 CNY [4,195.02, 20,440.44]. A total of 17,235 patients (20.0%) underwent surgery. Among the patients, 434 (0.5%) experienced HAIs, predominantly respiratory infections, accounting for 284 cases (65.44%). The distribution and composition of device-associated infections were as follows: catheter-associated urinary tract infections (9 cases, 2.07%), central line-associated bloodstream infections (5 cases, 1.15%), and ventilator-associated pneumonia (3 cases, 0.69%).

A total of 115 cases were identified as MDRO infections. The primary MDRO infection types included:

- Methicillin-resistant *Staphylococcus aureus* (MRSA): 47 cases.
- Carbapenem-resistant *Pseudomonas aeruginosa* (CR-PA): 29 cases.
- Carbapenem-resistant *Acinetobacter baumannii* (CR-AB): 20 cases.
- Carbapenem-resistant Enterobacteriaceae (CRE): 18 cases.
- Vancomycin-resistant Enterococcus (VRE): 1 case.

Among these patients with MDRO infections, 95 cases (82.61%) had already been infected with MDRO before hospitalization, among which MRSA was mainly infected before admission (42 cases, 89.36%). There were 21 patients with nosocomial infection of MDRO, and the types of those were mainly CR-AB (8 cases, 38.10%), CR-PA (6 cases, 25.57%), and CRE (2 cases, 9.52%).

Regarding invasive device usage, 2,476 patients required mechanical ventilation, with a median duration of 2 days [1, 2]. A total of 51,520 patients underwent central venous catheterization, with a median duration of 5 days [3, 8], and 15,186 patients required urinary catheterization, with a median catheter duration of 2 days [2, 4]. Additional general information is provided in Table 1.

**Table 1:** Demographic and clinical characteristics of patients.

Variable	Group	Number of cases and proportion (%)
Gender	Female	45,617 (52.93%)
	Male	40,560 (47.07%)
Surgical Status	No	68,942 (80.00%)
	Yes	17,235 (20.00%)
MDRO Infections	No	86,062 (99.87%)
	Yes	115 (0.13%)
MDRO Infection Types	CR-AB	20 (17.54%)
	CR-PA	29 (25.44%)
	CRE	18 (15.79%)
	MRSA	47 (41.23%)
	VRE	1 (0.09%)
HAI Status	No	85,743 (99.5%)
	Yes	434 (0.50%)
Disease categories	Lung cancer	16,608 (19.27%)
	Breast cancer	10,555 (12.25%)
	Cervical cancer	5,863 (6.80%)
	Rectal cancer	4,464 (5.18%)
	Esophageal cancer	4,452 (5.17%)
	Nasopharyngeal cancer	3,558 (4.16%)
	Thyroid cancer	3,587 (4.16%)
	Colon cancer	3,465 (4.02%)
	Gastric cancer	3,372 (3.91%)
	Ovarian cancer	3,277 (3.80%)
	Non-specific malignant tumors	3,338 (3.87%)
	Liver and intrahepatic bile duct cancer	2,089 (2.42%)
	Others	20,809 (31.83%)
Infection sites	Respiratory system	284 (65.44%)
	Circulatory system	48 (11.06%)
	Digestive system and abdominal region	44 (10.14%)
	Surgical sites	32 (7.37%)
	Urinary system	18 (4.15%)
	Skin and soft tissues	3 (0.69%)
	Others	5 (1.15%)

HAI: hospital-acquired infection; MDRO: multidrug-resistant bacteria.

### 3.2 Propensity score matching for patients with hospital-acquired infections

As there were only 434 cases of HAIs among the study population, PSM was employed to address the disparity in sample sizes between HAIs and non-HAI patients to ensure comparability. Patient baseline characteristics and the specific nature of HAIs were comprehensively considered. Age and gender were used as basic indicators. Preoperative NLR, an inflammatory biomarker, was included as it reflects systemic inflammation levels. Recent studies have highlighted the critical role of systemic inflammation in the onset and progression of cancer, and NLR has shown predictive value for early diagnosis, prognosis, and recurrence in cancer patients (11–14).

**Figure 1:**
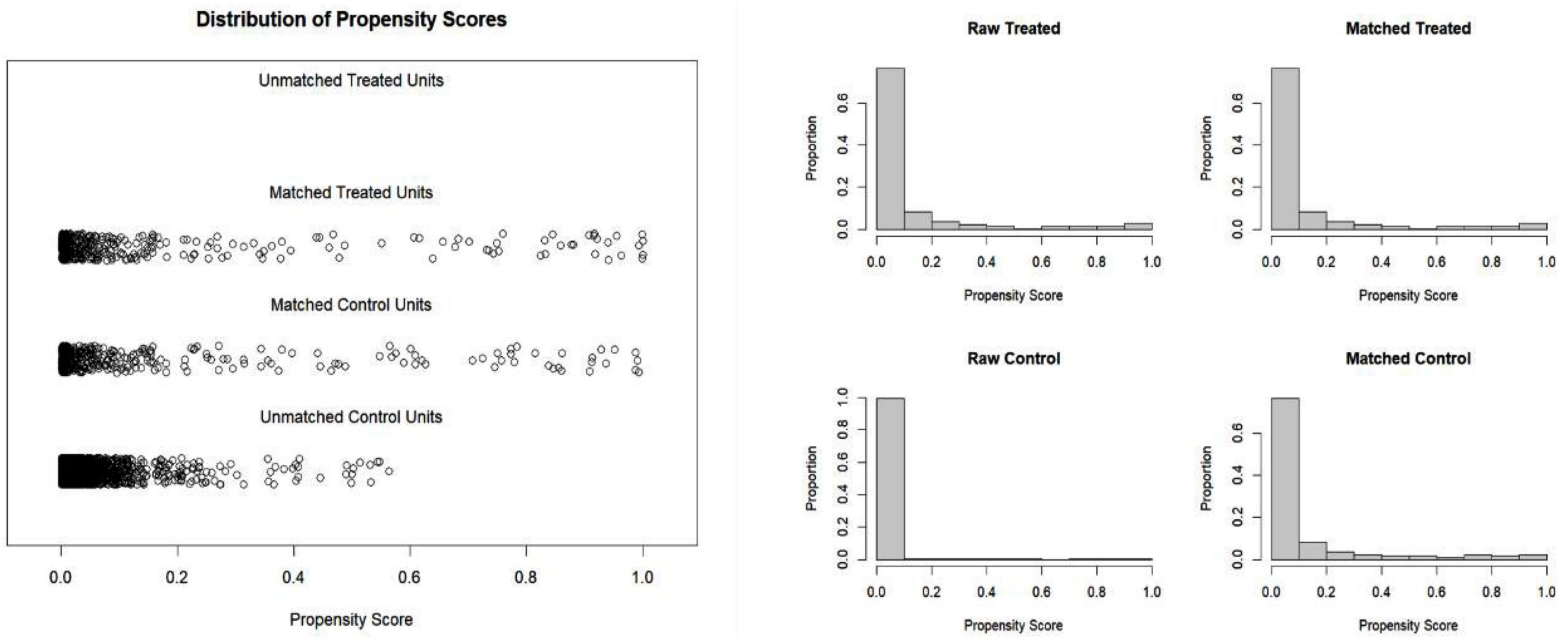
**(A)** The propensity value before and after the matching of nosocomial infections. **(B)** The histogram of propensity value of the patients with nosocomial infections before and after the matching.

Furthermore, HAIs are often associated with factors such as surgery, mechanical ventilation, central venous catheterization, and urinary catheterization (15–18). Therefore, the matching variables included age, gender, NLR, surgical status, days of mechanical ventilation, days of central venous catheterization, and days of urinary catheterization. Using these variables, 1:1 PSM was performed, resulting in a matched dataset of all 434 HAI patients paired with 434 non-HAI patients, yielding a total sample size of 868 patients. After matching, the propensity score distributions of the HAIs and control groups were nearly identical (Figure 1). The balance of variables such as gender, age, and NLR between the two groups was significantly improved after matching (Table 2).

**Table 2:** The balance test of the Covariates.

Variable	Before Matching			SMD^*^	After Matching			SMD^*^
	Control (*n* = 77,214)	HAIs (*n* = 434)	*p*		Control (*n* = 434)	HAIs (*n* = 434)	*p*	
Gender (*n*, %)			<0.001	0.402				
Male	35,423 (45.90)	284 (65.40)			308 (71.00)	284 (65.40)	0.094	0.119
Female	41,791 (54.10)	150 (34.60)			126 (29.00)	150 (34.60)		
Age (years, mean ± SD)	55.61 ± 13.01	59.54 ± 15.13	<0.001	0.279	60.05 ± 13.19	59.54 ± 15.13	0.599	0.036
NLR (mean ± SD)	88.71 ± 95.88	113.46 ± 320.63	<0.001	0.105	107.87 ± 235.02	113.46 ± 320.63	0.770	0.020
Surgery (*n*, %)			<0.001	0.581			0.309	0.074
Yes	16,648 (21.60)	209 (48.20)			225 (51.80)	209 (48.20)		
No	60,566 (78.40)	225 (51.80)			209 (48.20)	225 (51.80)		
Mechanical Ventilation Days (mean ± SD)	0.05 ± 0.43	1.53 ± 3.64	<0.001	0.568	1.15 ± 2.98	1.53 ± 3.64	0.095	0.114
Central Venous Catheter Days (mean ± SD)	3.80 ± 4.60	15.97 ± 13.88	<0.001	1.177	15.65 ± 13.17	15.97 ± 13.88	0.733	0.023
Urinary Catheter Days (mean ± SD)	0.68 ± 1.97	7.03 ± 9.51	<0.001	0.924	6.29 ± 9.09	7.03 ± 9.51	0.240	0.080

*Standardized Mean Difference; SD: Standard Deviation.

### 3.3 Hospital infection disease burden calculation

After propensity score matching, a permutation test was applied to compare hospitalization costs and length of stay (LOS) between the hospital infection group and the control group. The results (Table 3) showed that both hospitalization costs and LOS were statistically different between the hospital infection group and the control group (*p* < 0.05). The hospital infection group had higher hospitalization costs (median difference of 12,417.60 yuan, a 23.70% increase) and longer LOS (median difference of 5.50 days, a 33.30% increase) compared to the control group.

**Table 3:** The permutation test of patients with Non-HAIs and HAIs.

Variable	Non-HAIs [p25-p75]	HAIs [p25-p75]	Median Difference (95%CI)	Change Rate (%)	Z	*p*
Hospitalization Costs (Yuan)	52382.50 [20151.7, 81615.9]	64800.10 [34379.5, 98573.2]	12417.60 (4442.82, 20209.02)	23.70%	−4.316	<0.001
Length of Stay (Days)	16.50 [10.00, 25.00]	22.00 [15.00, 28.30]	5.50 (4.00, 7.00)	33.30%	−4.177	<0.001
Medicine Cost	10029.79 [3499.38, 21827.23]	15483.58 [7490.91, 28487.25]	5453.79 (2612.75, 7866.08)	54.38%	−3.295	<0.001
Material Cost	9064.22 [943.39, 21059.36]	12598.26 [2819.52, 28139.96]	3534.04 (−321.26, 7452.13)	38.99%	−4.176	<0.001
Laboratory Testing Cost	8596.00 [4300.00, 13795.00]	11226.50 [6674.00, 16631.00]	2630.50 (1531.68,3837.07)	30.60%	−5.056	<0.001
Surgery-Related Cost	6748.02 [55.00, 11066.00]	8532.87 [2271.00, 13189.43]	1784.85 (820.37, 3484.99)	26.44%	−3.236	<0.001
Treatment Cost	4393.45 [1678.00, 10184.40]	5754.72 [3253.55, 11559.50]	1361.27 (277.02, 2206.48)	30.98%	−1.795	0.036
Nursing Cost	1826.50 [975.00, 3156.00]	2382.50 [1571.00, 3792.00]	556.00 (359.22, 787.36)	30.44%	−1.826	0.034
Traditional Medicine Cost*	0 [0, 72.11]	0.32 [0, 110.00]	0.32 (0.00, 12.56)	/	−0.604	0.273

*Indicates small data values for the item. HAIs: hospital-acquired infections.

To further investigate the specific cost differences in hospitalization expenses between the hospital infection group and the control group, a permutation test was conducted for various cost categories. The results (Table 3) are as follows.

The permutation test results revealed statistically significant differences in material costs, treatment costs, medicine costs, laboratory testing costs, nursing costs, and surgery-related costs between the hospital infection group and the control group. Among these, the largest difference was observed in western medicine costs, with a median difference of 5,453.79 Yuan (change rate: 54.38%). This was followed by material costs, with a median difference of 3,534.04 Yuan (change rate: 38.99%).

### 3.4 Calculation of disease burden from multidrug-resistant infections

To explore the disease burden caused by MDRO bacterial infections, the study sample, hospital infection group, and control group were divided into MDRO infection groups and non-MDRO infection groups. The differences in hospitalization costs and lengths of stay among the three groups were compared. Results are shown in Table 4. Permutation test results indicate that MDRO infections significantly increase hospitalization costs and LOS in the study population.

**Table 4:** Comparisons of the overall hospital costs and stays between Patients with MDRO and with Non-MDRO.

Group	Non-MDRO*[p25, p75]	MDRO [p25, p75]	Median Difference (95%CI)	Change Rate (%)	Z	*p*
Cost						
All Patients	57178.09 (27528.34, 88556.64)	89444.71 (58269.49, 144504.36)	32266.62 (7,651, 81,434)	56.43%	−2.921	0.002
HAIs	63988.27 (33545.67, 97179.72)	89444.71 (58583.76, 144504.36)	25456.44 (2,500, 80,125)	39.78%	−1.909	0.028
Non-HAIs	52015.35 (20276.27, 81440.71)	76311.64 (34331.56, 166786.06)	24296.29 (−43,899, 188,900)	46.71%	−1.513	0.065
Days						
All Patients	19 (13, 26)	27 (20, 41)	8 (−1, 14)	42.11%	−3.514	<0.001
HAIs	21 (15, 28)	27 (20, 41)	6 (−2, 14)	28.57%	−2.489	0.006
Non-HAIs	16 (10, 25)	35.50 (18, 50)	19.50 (−8, 40)	121.88%	−2.144	0.016

*MDRO: multidrug-resistant bacteria.

## 4 Discussion

### 4.1 Increased disease burden caused by hospital-acquired infections and multidrug-resistant infections

Previous studies have demonstrated that HAIs contribute to a greater disease burden, characterized by increased hospitalization costs and LOS (19–21). This study, focused on an oncology specialty hospital, compared the hospitalization costs and LOS of patients with HAIs and non-HAI. The findings revealed that hospitalization costs in the HAIs were 23.70% higher than non-HAI, with a median difference of 12,417.6 CNY. Hospitalization costs in the HAIs group were 1.24 times those of the non-HAI group, consistent with previous studies reporting cost differences of 1.32 and 1.49 times (22, 23). Additionally, LOS increased by 33.30%, with a median difference of 5.50 days, demonstrating that HAIs necessitate longer hospital stays and higher economic costs, aligning with earlier research conclusions.

Patients with MDRO infections incurred 56.43% higher hospitalization costs and a 42.11% longer LOS compared to non-MDRO patients, indicating a substantial increase in disease burden due to MDRO infections. In terms of specific costs, this study, unlike previous studies that directly examined total hospitalization costs associated with HAIs (24, 25), further analyzed individual cost components. It identified western medicine expenses as the largest cost discrepancy, with the median cost in the HAI group reaching 15,483.58 CNY—1.5 times that of the control group. This result may reflect the association between antimicrobial drug usage and HAI treatment. The costs for materials, treatment, laboratory tests, nursing, and surgery-related in the HAIs group were all higher than those in the control group. Specifically, the material costs in the infection group were approximately 1.39 times those of the control group, and the laboratory test costs were 1.31 times higher. These increased expenses may be attributed to the need for additional examinations, treatments, nursing care, and even surgeries, necessary both to address the hospital-acquired infections and related complications and to mitigate the resultant physical damage and risk of exacerbating patients’ pre-existing conditions (26–28).

.In clinical practice, when patients develop HAIs, pathogenic microorganisms such as bacteria or MDRO often trigger inflammation, fever, and complications. To alleviate and relieve these symptoms, the use of various antibiotic drugs is one of the important treatment modalities (29, 30). However, many clinicians report challenges in appropriately selecting these antibiotics and acknowledge insufficient knowledge, which not only increases medical expenses but also exacerbates bacterial resistance (31, 32). Moreover, for hospitals, higher hospitalization costs and longer LOS result in lower bed turnover rates and diminished operational efficiency, which adversely affect the hospital’s sustainable operations (33). These challenges also create added financial pressure on medical insurance systems, contributing to the indirect economic burden of HAIs and MDRO infections (34). Therefore, for hospitals and patients, it is extremely urgent to prevent and reduce HAIs and MDRO infections. Hospitals can establish a real-time detection system for antibiotic use, combine it with electronic medical record data, dynamically adjust the medication regimen (35), Conduct regular antibiotic stewardship training for clinical departments, providing updated antimicrobial susceptibility summary reports and trend analyses to ensure proper antimicrobial use in clinical practice.; strengthen the knowledge education and behavior specification management of infection prevention and control for medical staff and cleaning staff. In particular, it is necessary to improve the compliance of medical staff with hand hygiene to reduce the risk of cross-infection; implement contact isolation for patients colonized or infected with MDRO to cut off the transmission chain, so as to strengthen the management of antibiotic use and the prevention of hospital-acquired infections.

### 4.2 Establishing a model for measuring the disease burden of HAIs and MDRO infections

In hospitalized patient populations, the proportion of HAIs and MDRO infection patients is typically small. This study included 434 HAIs and 114 MDRO infection patients. To enhance statistical power in small sample sizes, minimize the influence of confounding factors, and improve result reliability, we combined PSM and permutation testing to estimate the disease burden of HAIs and MDRO infections.

PSM maximizes data utilization, reduces confounding effects, and enhances group comparability (36, 37). Following matching, key variables were better balanced between groups, enabling precise evaluation of the impact of infections on hospitalization costs and LOS. This process also improved sample representativeness, strengthening the credibility of the findings.

Given the skewed distribution of the data in this study, traditional parametric methods were unsuitable. Permutation testing, a nonparametric approach, does not rely on distribution assumptions. It yields robust results for skewed data on hospitalization costs and LOS, allowing accurate estimation of the economic burden and LOS impact associated with HAIs and MDRO infections. At the same time, the calculation method of combining PSM with permutation test adopted in this study has improved the representativeness of the samples and enhanced the credibility of the research results. It is worth noting that the European Centre for Disease Prevention and Control has advocated the use of advanced statistical methods such as PSM for the assessment of attributable mortality and excess length of stay in patients with ICU-acquired infections (38) This coincides with our research method and demonstrates how the data-driven model can effectively bridge the gap between local clinical practices and international benchmarking work.

### 4.3 Clinical and health policy implications

HAIs and MDRO infections endanger cancer patients with weakened immunity and increase financial burdens on patients and their families. Irrational antimicrobial use promotes resistance and hinders recovery. This study highlights the significant impact of HAIs on hospitalization costs and LOS for cancer patients, underscoring the need for stronger prevention and intervention measures. Prioritizing rational antimicrobial use and MDRO infection control is crucial to reducing extended hospital stays and additional costs associated with these conditions. Additionally, the combination of PSM and permutation testing used in this study offers a valuable tool for evaluating the effectiveness of infection control strategies in reducing the disease burden (39). This approach can guide policymakers in designing more targeted and effective prevention measures in the future. From a policy perspective, the WHO Global Action Plan on Antimicrobial Resistance (2015) and China National Action Plan to Contain Antimicrobial Resistance (2022–2025) both prioritize reducing MDRO infections through stricter antimicrobial use regulations and enhanced infection control infrastructure. Our results on the disproportionate rise in western medicine costs (54.38%) underscore the necessity of aligning hospital-level antimicrobial stewardship with these national and global agendas. Currently implemented infection prevention and control policies in China, such as *the Technical Guideline for the Prevention and Control of MDRO Infections in Healthcare Settings (Trial Implementation)*, *Technical Guideline for the Prevention and Control of Surgical Site Infections (Trial Implementation)*, *Technical Guideline for the Prevention and Control of Catheter-Associated Bloodstream Infections (Trial Implementation),* etc., collectively underscore the critical importance of both personnel and institutional management in combating HAIs.

The effective implementation and operationalization of these policies are fundamentally dependent on robust financial backing and managerial commitment. Financially, adequate funding must be allocated to support hospital infection management. This includes resources for incentivizing clinical and infection control personnel, procuring necessary equipment, training programs, etc. Such investment is essential to ensure sufficient human, material, and financial resources are available for infection control activities. Managerially, high-level prioritization of infection prevention and control is imperative. This necessitates establishing clear organizational structures and accountability frameworks, leveraging information technology for enhanced capabilities, The ultimate goal is to translate policies into consistent, ingrained practices within daily clinical routines.

## 5 Limitations and future directions

This study’s reliance on single-center data limits external validity, and future studies should adopt a multicenter design. Although PSM reduces known confounders, unmeasured factors, such as treatment regimens, anti-infection variations, and economic conditions, may still influence outcomes. Future research should extend to multiple oncology hospitals to validate the applicability of combining PSM and permutation testing, thereby broadening the findings’ generalizability.

Additionally, integrating advanced statistical methods, such as propensity score weighting and generalized estimating equations, could further refine disease burden assessments and enhance analytical depth. Further exploration of infection risks and burden disparities across cancer types is warranted to provide a foundation for more precise and targeted preventive strategies.

## 6 Conclusion

This study innovatively employs PSM and permutation testing to provide more precise estimates of the impact of HAIs and MDRO infections on the disease burden of cancer patients, offering important evidence for clinical and policy practices. For the hospital infection control department, the results of the increased economic burden caused by HAIs and MDRO can provide data support for obtaining more funds for the prevention and control of hospital infections. In terms of policies, in recent years, in order to address the issue of microbial resistance, since 2016, China has issued and implemented the National Action Plan for Containing Microbial Resistance (2016–2020) and the National Action Plan for Containing Microbial Resistance (2022–2025). The comprehensive strategy for containing drug resistance has achieved positive results. However, the drug resistance problems of some common microorganisms are still worsening, and there are disparities in the level of drug resistance prevention and control among regions and institutions (40, 41). The prevention and control of infections in medical institutions and the management of the clinical application of antimicrobial drugs should continue to be promoted in a coordinated manner, and more investment should be made in infection prevention and control work, including the allocation of infection control professionals and the construction of infection control technical capabilities. In terms of hospital management, various infection prevention and control systems, norms and standards should be actively implemented. Daily training for medical staff on the rational application of antimicrobial drugs and the prevention and control of resistance should be strengthened to improve the theoretical knowledge and practical skills of medical staff related to the prevention and control of MDRO. Implement the Measures for the Management of the Clinical Application of Antibacterial Drugs, regularly train and assess physicians and pharmacists. Those who pass the assessment shall be granted the corresponding prescription rights for antibacterial drugs or the qualifications for dispensing antibacterial drugs. Guided by the research results, the incidence of hospital-acquired infections and infections with drug-resistant bacteria in medical institutions should be reduced, the source classification of medical waste should be strengthened, and the economic burden caused by HAIs should be reduced from the source. While previous studies have focused on general hospitals, this research centers on specialized cancer hospital, designing a model for calculating the disease burden of HAIs and MDRO infections for these specialized facilities, and providing insights and strategies for the prevention and control of HAIs and MDRO infections in specialized cancer hospital. In the future, this model for estimating the burden of infection can be validated in more specialized cancer hospitals, especially those in different regions with varying scales and resource levels, to improve the generalizability of the results. The standardization of HAIs detection data should be further promoted, and a database sharing platform should be established and developed to facilitate the integration and analysis of multi-center data, and to promote the globalization of research on the prevention and control of HAIs and MDRO infections.

## Data Availability

The raw data supporting the conclusions of this article will be made available by the authors, without undue reservation.
